# Developing interactive VR-based digital therapeutics for Acceptance and Commitment Therapy (ACT): a structured framework for the digital transformation integrating gamification and multimodal arts

**DOI:** 10.3389/fpsyt.2025.1554394

**Published:** 2025-06-04

**Authors:** Hyungsook Kim, Yoonyoung Choi

**Affiliations:** ^1^ HY Digital Healthcare Center, Hanyang University, Seoul, Republic of Korea; ^2^ Department of Data Science, Hanyang University, Seoul, Republic of Korea

**Keywords:** digital therapeutics (DTx), interactive VR, digital transformation, Acceptance and Commitment Therapy (ACT), multimodal arts guidance, gamification, human-computer interaction (HCI)

## Abstract

**Introduction:**

Digital therapeutics (DTx) require structured methodologies to translate evidence-based psychotherapy into immersive digital formats. In response to this need, this study proposes a practical framework for the digital transformation of Acceptance and Commitment Therapy (ACT) into an interactive virtual reality (VR) system.

**Methods:**

DTx-ACT, designed as a therapeutic intervention for depression, is a VR-based system that delivers ACT through an immersive virtual experience. Its development followed five structured phases: preliminary research, design, development, advancement, and commercialization. The original ACT protocol was modularized into VR environments using the Session Structuring System (SSS) model. To enhance user engagement, gamification and multimodal arts strategies were incorporated. As part of the development process, evaluation metrics were defined to assess both clinical effectiveness and user interaction.

**Results:**

The final system comprises five immersive VR sessions, each lasting 6 to 12 minutes. These modules incorporate ACT metaphors, interactive tasks, and multisensory feedback to enhance therapeutic engagement. To support the digital transformation of ACT, three core components were established: (1) an evidence-based therapeutic protocol, (2) interactive VR elements—including gamification and multimodal arts-based guidance, and (3) a data-driven evaluation framework. Evaluation metrics, derived from a pilot study, were integrated into the system, which collects clinical and interaction data—such as real-time behavioral patterns and sensor-based information—to enable comprehensive evaluation.

**Discussion:**

Based on this development process, we propose a practical framework for designing interactive VR-based DTx. This framework bridges clinical structure, creative engagement, and real-time evaluation to support personalized and scalable applications in digital mental healthcare. It contributes to the standardization of digital transformation in evidence-based therapy and offers a transferable model for future therapeutic content development.

## 1 Introduction

In recent years, the fields of arts, psychology, and mental health care have experienced continuous innovation through digital technologies (1). Amid the growing prevalence of mood disorders—notably depression and anxiety—digital media has emerged as a promising platform for delivering psychological interventions (2–4). This shift has accelerated the development of digital therapeutics (DTx), which deliver clinically validated mental health treatments via software-based interventions.

Recognizing DTx’s potential, regulatory agencies including the U.S. Food and Drug Administration (FDA), the UK’s National Institute for Health and Care Excellence (NICE), and Korea’s Ministry of Food and Drug Safety (MFDS) have established official certification pathways. In 2024, the FDA approved Rejoyn and DaylightRx as the first prescription DTx for major depressive disorder (MDD) and generalized anxiety disorder (GAD), respectively (5–9). NICE has conditionally recommended interventions such as Beating the Blues and SilverCloud (10–12), while Korea’s MFDS approved Somzz for insomnia and issued regulatory guidelines to support digital health innovation (13–15). Most of these products are grounded in Cognitive Behavioral Therapy (CBT) and have undergone rigorous validation for both software design and therapeutic efficacy.

As digital health technologies evolve within the Fourth Industrial Revolution, emerging platforms such as virtual reality (VR) and extended reality (XR) are opening new frontiers for mental health interventions (16). A growing body of research has demonstrated the clinical effectiveness of VR in treating psychiatric conditions and promoting adherence to digital interventions for depression, thereby reinforcing its therapeutic potential in mental healthcare (17–19). VR enables immersive, multisensory environments that simulate therapeutic contexts. When combined with gamification and multimodal arts—including visual, musical, and interactive modalities—these technologies can enhance user engagement, emotional immersion, and therapeutic adherence (20–25).

However, several essential components must be established to ensure the effectiveness of digital mental health interventions. First, unlike face-to-face therapy, which allows for clinical intuition and flexibility, digital interventions require standardized procedures and structured session designs to ensure treatment fidelity and reproducibility (26–29). Therefore, a systematic methodology is needed to translate evidence-based psychotherapies into interactive digital formats while preserving their core therapeutic mechanisms. Second, most existing DTx rely on mobile applications that provide passive user experiences, which limits active engagement and sustained motivation (30, 31). In contrast, interactive VR-based content provides a more immersive and engaging alternative that can foster deeper therapeutic involvement. Third, there is a lack of practical frameworks for designing immersive therapeutic environments that effectively integrate arts-based and gamified components. Advancing such frameworks is essential to enhance both user experience and the clinical rigor of VR-based DTx.

To address these challenges, this study proposes a structured, clinically grounded framework for developing interactive VR-based DTx guided by Acceptance and Commitment Therapy (ACT). This framework contributes to the field in three key ways: it presents a systematic methodology for digitalizing ACT protocols; it outlines actionable design principles for integrating multimodal arts and gamification into immersive VR therapy; and it provides a reproducible process to guide content development. Unlike previous studies that focused on standalone content or mobile-based formats, our approach clearly defines how to implement and integrate evidence-based practices (EBPs) within immersive environments. These environments are enhanced by multimodal arts and gamification principles, improving the validity and scalability of digital mental health interventions.

## 2 The conceptual framework for the development of DTx-ACT

This section introduces the conceptual framework for developing DTx-ACT, an interactive VR-based digital therapeutic system designed to deliver ACT for depression. The framework consists of two key components: a structural protocol for digital transformation and design principles for interactive VR-based interventions.

### 2.1 A structural protocol for the digital transformation of ACT

The development of DTx can be achieved by systematically translating well-established EBPs into digital formats. In this study, ACT was selected as the theoretical foundation for this transformation, and the structural protocol for developing DTx-ACT was structured around three core elements.

First, the Program Logic Model (PLM) was utilized to establish a high-level conceptual roadmap for the system’s development. While PLM is commonly used to organize complex interventions in healthcare, education, and social services (32–34), its role in this study was primarily to guide initial planning and structural design, rather than detailed content analysis. The PLM-based framework for system development is outlined in **Table 1**.

**Table 1:** Development roadmap of interactive VR-based DTx for ACT (DTx-ACT).

Interactive VR-based DTx development roadmap			
Input (Resources) →	Activity (Design) →	Output (Development) →	Outcome (Evaluation)
Original evidence-based practices (EBPs) • Acceptance and commitment therapy (ACT): protocol and manual • Validated digital interventions for mental health	Digital transformation for ACT • Session Structuring System (SSS) analysis • Gamification and multimodal arts guidance design	Development of interactive VR-based digital therapeutics for ACT (DTx-ACT) • Five-session immersive VR modules • Data-driven evaluation System	Validation of efficacy and preparation for regulatory approval • Analysis of clinical and interaction data • Regulatory approval process
Traditional method (TM)	→	Digital transformation (DT)	

The symbol “→” in **Table 1** represents flow and procedural sequence within the framework. It indicates progression from one phase to the next (e.g., Input to Activity, Traditional method to Digital transformation).

Second, ACT was chosen based on its demonstrated clinical effectiveness in treating depression and its structured implementation as an evidence-based psychotherapy. Meta-analytic evidence supports ACT’s efficacy (35–38), particularly through mechanisms that target experiential avoidance and psychological inflexibility (39). Additionally, ACT’s phased treatment structure and standardized session components (introduction, core intervention, and conclusion) provide a modular foundation well suited for digital adaptation.

Third, the Session Structuring System (SSS) model was applied to operationalize the ACT protocol within immersive VR environments. Originally developed for integrative arts therapy (40, 41) and later adapted for public mental health contexts (42), the SSS supports both macro- and micro-level design. At the macro level (i.e., comprehensive whole sessions), it defines session goals, duration, activities, and evaluation methods. At the micro level (i.e., respective single sessions), it structures the therapeutic flow, timing, and environmental configuration. This framework enabled the development of modular, immersive VR sessions that balance clinical rigor with user-centered flexibility.

### 2.2 Development principles for interactive VR-based DTx

#### 2.2.1 Therapeutic effects of VR-based interventions

Medical XR technologies, including AR (augmented reality) and VR, have the potential to enhance healthcare services by providing novel treatment modalities and tools for diagnosing and treating health conditions (43). These technologies have transformed healthcare delivery by extending clinical services, which are typically confined to hospitals and clinics, directly to patients in their homes or other non-clinical environments. Notably, VR has emerged as an exceptionally promising technology that has rapidly attracted attention for the treatment of depression and anxiety (44, 45).

VR utilizes wearable devices, such as head-mounted displays (HMDs), to transport users into a three-dimensional virtual space, providing sensory experiences based on presence and immersion to facilitate interaction. Immersive VR integrates digital technologies, including graphics, images, videos, and sounds, to perceive the user space and enable identical actions in the VR world. Moreover, VR devices include sensors that detect user movement and rotation, allowing for realistic responses and collection of user data such as movement and gaze data. These features enhance the functionality of VR as a digital therapeutic tool, enabling personalized treatment processes and offering adaptability to individual patient needs.

Building on these technological advancements, recent systematic reviews indicate that VR-based interventions are effective in managing various mental health conditions, including depression, anxiety, and post-traumatic stress disorder (PTSD) (22). A further review highlighted that VR enhances emotional engagement and reduces avoidance behaviors, particularly when combined with EBPs such as CBT and mindfulness (46). Furthermore, a recent meta-analysis focusing on VR and gamification-based interventions confirmed their consistent benefits in improving treatment engagement and alleviating symptoms of depression and anxiety (20). While these findings are promising, the reviews also emphasize the need for larger-scale randomized controlled trials to strengthen the clinical evidence base.

These findings are evident in real-world clinical applications. For example, Oxford VR has successfully automated VR psychotherapy for patients with mental disorders using gameChangeVR technology, offering CBT in a secure and immersive VR environment across six sessions over six weeks (47). Such approach has shown promise in assisting individuals with schizophrenia and mood disorders who experience psychotic symptoms and face anxiety and distress in their daily lives (48, 49). In addition, RelieVRx, the FDA approved VR digital therapy for chronic pain relief, incorporates gamification principles based on evidence from CBT, mindfulness, and pain neuroscience education to validate the efficacy of VR treatment (50). Patients are able to self-administer VR therapy comfortably at home, with sessions averaging 6 min each, totaling 56 sessions overall. Furthermore, the Blue Room, CBT-based VR therapy, has demonstrated clinical effectiveness in treating social phobia in children with autism (51).

Given these successful applications, the potential of VR to revolutionize mental health treatment is considerable. Moreover, this growing body of clinical validation underscores the feasibility of VR technology as a viable digital therapeutic tool. Building on these findings, we selected VR as the medium for developing evidence-based DTx.

#### 2.2.2 Gamification strategies for enhancing mental health interventions

Recent clinical studies have demonstrated that therapeutic software incorporating gamification elements offers significant therapeutic benefits for managing depression. Gamification in the healthcare sector is expected to motivate individuals to improve their lifestyle habits and health outcomes and maintain these changes over time (52). Gamification involves the strategic integration of gaming elements into conventional activities or processes to enhance user engagement, motivation, learning, or interaction. This entails infusing non-game contexts with elements that resemble those found in games to enrich user experiences and encourage desired behaviors, ultimately prompting specific actions.

Software applications that apply gamification, such as Alcor, Beating the Blues, DAHLIA, Deprexis, and E-couch, have been clinically validated to demonstrate their therapeutic effects on depression (12, 52–57). The application of games for digital mental health interventions, such as the aforementioned ones, can be developed by considering key factors, such as engagement, accessibility, consistency with treatment, generalizability, and effective skill building (58) (**Table 2**). In addition, research on gamification principles for internet interventions (71) suggests that these principles encompass various elements such as supporting player archetypes, meaningful choices, meaningful purposes, feedback, and visibility of progress. These principles can be classified into four main categories: system design and use, user characteristics, environmental factors, and translational aspects.

**Table 2:** Guidelines for digital mental health game development (58).

Key factors	Guidelines	References
Engagement	• Characters and gameplay should relate to the patient. • Gameplay must be motivating, interesting, and engaging. • Length of gameplay must be not too long or short. • Game must have immediate feedback. • Game must be easy to play. • Game should have a large base of novel stimuli or situations.	(59–64)
Accessibility	• Game must be easily accessible. • The game must work in the treatment environment.	(59, 61, 64–67)
Consistency with treatment	• Develop with your patient in mind. • Game design and play should be conceptually sound, in terms of best practices for both therapy and training. • Game must have clearly defined and implemented learning goals.	(59, 68, 69)
Generalizability	• Game must conform to a high degree of realism and immersion. • Too much or too little input of the player can affect the usefulness of the intervention. • Game content has real-world problems.	(59–61, 63, 70)
Effective skill building	• For true skill development to occur, a game-based environment must have the learner work through the three stages of skill acquisition: cognitive, associative, and autonomous. • Gameplay must be frequent yet not excessive. • Game must be interactive with moderate task demand.	(59, 60, 63, 64)
General advice for developers	• Game designers should be well versed in subject matter and videogames.	(59)

In this study, we applied gamification principles to the development of interactive VR therapeutics. The core components of DTx-ACT were analyzed based on four key elements: user characteristics, environment, system design, and translational aspects. Our design and implementation process incorporated considerations for engagement, accessibility, consistency with treatment protocols, generalizability, and effective skill-building. The integration of gamification elements was strategically employed to sustain user interaction and motivation. This approach ensured that DTx-ACT was designed to be user-friendly while actively promoting user engagement.

#### 2.2.3 Multimodal arts for immersive and multisensory engagement

VR environments provide a multidimensional sense of engagement by integrating visual, auditory, and kinesthetic stimuli. This immersive experience enhances emotional processing and has the potential to evoke intense emotional responses. Research suggests that psychological and physical well-being is fostered through emotional processing mechanisms, where bodily sensations are recognized, accepted as interpretable data, and transformed into emotional constructs expressed symbolically and linguistically. Furthermore, multisensory stimulation—encompassing visual, auditory, and tactile inputs—has been shown to aid in mood regulation and alleviate symptoms of depression. Artistic guidelines can also serve as effective sensory tools to facilitate emotional processing.

When managing depression, the possible benefits of integrating multiple senses should be considered instead of depending on a single modality. Research has shown that listening to music can help reduce feelings of depression and anxiety (72–76). Additionally, the combination of visual and aural cues enhances participants’ emotional experiences (44), ultimately resulting in positive effects on depression and anxiety (44, 77). In addition, the variety of sensory stimuli in a credible VR environment enhances the players’ sense of presence. Multisensory stimulation can increase user engagement in VR environments, and scenarios that include visual, auditory, and passive tactile stimulation have been reported to positively affect presence in VR (78).

This study integrated various artistic elements with VR technology to enable interactive expression and appreciation within a three-dimensional space, aiming to enhance users’ emotional experiences. Recognizing the therapeutic value of multisensory integration, we developed immersive VR experiences as interactive content for psychological intervention. Our design incorporated multimodal arts-based guidance and gamification principles to promote engagement and improve therapeutic outcomes. By leveraging the distinct characteristics and techniques of diverse artistic media, we created interactive activities and metaphor-driven guidance grounded in evidence-based principles of ACT.

To systematize this process, we classified artistic elements into five genres: visual art, music, drama, literature, and dance/movement. This classification followed Choi’s (79) criteria, which analyzed the therapeutic functions of artistic media within the framework of integrative arts therapy (see **Table 3**). These criteria guided the translation of each genre’s therapeutic attributes into interactive techniques, enabling their integration as structured, experience-centered components of the VR intervention.

**Table 3:** Genre-specific therapeutic characteristics and functions in integrative arts therapy (41).

Genre	Therapeutic characteristics and functions
Art	- Expresses internal states through tactile and visual media - Symbolizes mental imagery through artistic forms - Enables personal and interpersonal communication through creative art - Utilizes nonverbal symbolic systems for therapeutic outcomes - Applies distancing techniques for interpretation and cognitive processing
Music	- Serves as a sensory-based sound medium - Provides dynamic experiences of rhythm, melody, and harmony - Induces pleasure and aesthetic experiences - Symbolizes mental imagery - Stimulates emotion and expands conscious awareness - Facilitates emotional catharsis and creative expression - Enhances communication through shared musical activity
Drama	- Incorporates verbal and physical movement - Expresses emotional and experiential content - Encourages role exploration as social beings - Evokes emotional and behavioral responses - Engages playful and cathartic processes - Stimulates spontaneity and creativity - Projects experience across real and virtual contexts - Enhances empathy and reflection through distancing techniques
Literary	- Visualizes the inner self through metaphor and symbolism - Expresses thoughts and emotions verbally - Reflects personal identity, character, and personality - Restructures memories to enable psychological release - Stimulates imagination and creative thought - Activates repressed desires and supports problem-solving - Facilitates self-reflection via narrative distancing
Dance Movement	- Channels dynamic energy through physical movement - Expresses unconscious desires through bodily action - Develops body image via somatic experiences - Delivers aesthetic experiences through creative movement - Reflects emotions and personality symbolically - Encourages interaction with the surrounding environment - Serves as a core medium for nonverbal communication

## 3 Methods

### 3.1 Study design

#### 3.1.1 Overview of study approach

This study was conducted as part of a national research and development (R&D) initiative in the Republic of Korea, aimed at developing DTx platforms for mental health management (80, 81). Its primary objective was to develop DTx-ACT—an interactive VR-based intervention for managing depression—and to establish a structured framework for designing such interventions.

Rather than evaluating clinical efficacy, the study adopted a conceptual and methodological approach, emphasizing the theoretical foundations and design strategies necessary for digital transformation. Specifically, we present DTx-ACT as an applied case to illustrate its development framework and design principles for interactive VR-based psychotherapy. This includes the adaptation of ACT—an evidence-based psychotherapy—into a VR-driven system, along with its theoretical rationale and implementation methods. While clinical validation lies beyond the scope of this study, the proposed framework provides practical guidance for future development and empirical research.

From the perspective of digital transformation in psychotherapy, this study explores how gamification principles and multimodal arts can be integrated into VR-based DTx. Digital transformation here refers to the systematic digitization of traditional psychotherapy, enabling standardized intervention design, automated data collection, and quantitative assessment. Accordingly, the development of DTx-ACT involved creating a framework that incorporates data-driven evaluation mechanisms within an immersive VR environment.

This study addresses two key research questions (RQs):

RQ1: What are the core components that guide the digital transformation of ACT into an interactive VR-based system?
RQ2: How are these components integrated into the design and implementation of the DTx-ACT system?

#### 3.1.2 Multidisciplinary Scrum team and development workflow

DTx-ACT was developed by a multidisciplinary Scrum team consisting of experts in digital healthcare, psychiatry, clinical psychology, human-computer interaction (HCI), and arts therapy. The content and clinical teams collaborated to analyze core ACT principles and to structure the VR-based intervention accordingly. To ensure conceptual coherence, regular focus group discussions were held to guide the development of therapeutic scripts that integrated ACT with gamification elements and multimodal arts strategies.

The system was implemented using the Meta Quest Pro headset, with immersive content developed in both Unreal Engine and Unity. Interaction dynamics, user interface (UI), and sensory immersion features were iteratively refined to enhance usability and user engagement. In parallel, a data acquisition system was designed in collaboration with data scientists to capture behavioral and sensor data—such as head movement and eye tracking—for future clinical evaluation.

#### 3.1.3 A pilot study for the evaluation framework

To evaluate the feasibility of the system’s evaluation framework, a pilot study was conducted during the study period (82). In preparation for developing DTx-ACT, we first designed and implemented a three-session VR module grounded in Mindfulness-Based Cognitive Therapy (VR-MBCT). This module was adopted due to its conceptual alignment with ACT, particularly in its emphasis on present-moment awareness, emotional acceptance, and cognitive flexibility. Given this theoretical proximity, the pilot study served as a practical foundation for assessing the viability of immersive delivery and multimodal evaluation strategies, which were subsequently integrated into the finalized DTx-ACT system.

The VR-MBCT module consisted of three structured sessions, each targeting a specific therapeutic process: constellation drawing for emotional exploration (Session 1), avatar-based perspective-taking (Session 2), and emotional labeling through symbolic interactions (Session 3). These sessions were developed in collaboration with clinical experts and delivered in an immersive VR environment.

Participants aged 18 to 40 were recruited through university counseling centers and online platforms, and were screened using the Patient Health Questionnaire-9 (PHQ-9). Based on their scores, participants were assigned to either the individuals with depression (IWD; n = 38) or individuals without depression (IWoD; n = 35) group. The study protocol consisted of three phases: (1) an introductory session involving informed consent and a VR interface tutorial, (2) an intervention session using the VR-MBCT module, and (3) a post-session review comprising standardized questionnaires and semi-structured interviews.

The system was implemented using the Meta Quest Pro headset, and electrodermal activity (EDA) was recorded via the Empatica E4 wristband. Multimodal data were collected to evaluate both interaction patterns and emotional responses, including gaze tracking, physiological arousal, and task-based behavioral logs. Statistical analyses were conducted using ANCOVA (controlling for age, gender, and prior VR experience), along with non-parametric tests (Wilcoxon signed-rank and Mann–Whitney U) to compare group-level differences. The insights gained from this pilot study directly informed the design logic, component selection, and data-driven evaluation strategy embedded in the DTx-ACT system.

### 3.2 Development process

#### 3.2.1 Preliminary research

In the preliminary research phase, we conducted a comprehensive market analysis of digital mental health services and reviewed existing studies to establish a solid foundation for our approach. This phase involved the following key steps:

First, we developed a digital transformation strategy based on evidence-based therapeutic approaches and appropriate media selection, guided by the SSS model. In relation to clinical factors, we compiled and reviewed clinical evidence to identify therapeutic protocols and assess their effectiveness, with particular focus on the core concepts and principles of ACT as an evidence-based psychotherapy.

Second, we organized the core components essential for developing evidence-based interactive VR therapeutics into three domains: clinical foundations, interactive VR elements, and data-driven evaluation mechanisms. For interactive VR elements, we examined how gamification and multimodal arts contribute to enhancing multisensory engagement and therapeutic efficacy. Based on these insights, we aimed to design interactive, arts-based interventions that integrate gamification principles into structured therapeutic scenarios. Regarding data-related components, we identified clinical indicators relevant to depressive symptoms to establish the foundation for a data-driven evaluation framework.

Third, we thoroughly examined the theoretical foundations and design features necessary for developing effective DTx. Through these foundational analyses, we established the conceptual and methodological basis for developing evidence-based interactive VR therapeutics, referred to as DTx-ACT.

#### 3.2.2 Design

In the design phase, we developed service scenarios, storyboards, and scripts while incorporating user interface and user experience (UI/UX) considerations. The therapeutic sequences were structured by identifying core elements from evidence-based psychotherapy protocols of ACT and translating them into therapeutic scenarios. To support this process, we proposed content outlines and project plans grounded in digital storytelling methodologies. Therapeutic scenes were constructed based on gamification principles and multimodal arts guidance, and scripts for interactive activities were developed in alignment with these designs. Additionally, we considered the therapeutic significance of each session and defined the types of data needed to extract meaningful insights, thereby laying the groundwork for subsequent data analysis.

#### 3.2.3 Development

In the development phase, the therapeutic scenarios structured in the previous phase were transformed into immersive VR content. To enhance therapeutic engagement, gamification principles were applied, and diverse artistic stimuli and guided interactions were incorporated. At the same time, user manuals were developed to guide operational procedures for delivering user-centered content. The development process followed an agile methodology to support iterative improvement of the content and therapeutic strategies.

To ensure clinical validation and regulatory readiness, DTx must undergo randomized controlled trials (RCTs) and incorporate digital biomarkers derived from real-time sensor and log data (83). To meet these requirements, we developed a data-driven evaluation system in parallel with content development and structured it around two core components. First, we created a framework to collect and analyze real-time data from DTx-ACT, including sensor outputs and user interaction logs, to support quantitative evaluation of therapeutic effects. Second, a clinical trial protocol for Software as a Medical Device (SaMD), based on the RCT model, was developed as part of the regulatory preparation for approval from the MFDS.

This study underscores the importance of clearly defining and operationalizing digital biomarkers as objective indicators for evaluating therapeutic outcomes. To support this goal, the evaluation framework was structured around two primary domains: clinical data and interaction data (e.g., digital biomarkers). This framework provides a systematic basis for categorizing and managing data types in future validation studies.

#### 3.2.4 Advancement

During the advancement phase, we refined the interactive VR therapeutic software developed in the preceding stages. The primary aim was to enhance system completeness and ensure conformity with medical device standards. Usability testing was conducted from a human–computer interaction (HCI) perspective within the context of digital healthcare. Based on user feedback, iterative modifications were made to continuously improve system functionality and user experience. In addition, clinical experts evaluated the content for its appropriateness for patients with depression and potential safety concerns. Their input informed content revisions to improve clinical applicability and reduce potential risks. This process supported the verification of the system’s core functionality and technical readiness. Therapeutic visualization components were also reviewed to promote engagement through immersive VR experiences. Revisions were implemented as necessary to maintain alignment with therapeutic guidelines and to ensure safety and clarity.

#### 3.2.5 Commercialization

This study presents a structured development methodology for DTx, with particular attention to regulatory considerations for classification as Software as a Medical Device (SaMD) under the MFDS (84). Although clinical trial results and formal approval procedures were beyond the scope of this study, regulatory readiness was addressed throughout the development process. During the study period, the research team submitted an application to the MFDS to determine the software’s eligibility as a medical device. Following regulatory review, it was classified as therapeutic software for the management of emotional disorders, confirming both its compliance with medical device standards and its potential for clinical application. In parallel, a patent application was filed to secure intellectual property rights. These regulatory and intellectual property measures formed essential groundwork for future clinical applications and technology transfer.

## 4 Results

The results consist of two parts, corresponding to RQ1 and RQ2. The first part presents the structural framework and principles guiding the digital transformation of evidence-based ACT. It focuses on three key elements: clinical evidence, interactive VR principles, and a data-driven evaluation system. This framework, derived from preliminary research, demonstrates how traditional ACT methodologies are adapted into a digital format. These findings address RQ1.

The second part outlines the design and implementation of the DTx-ACT system, emphasizing its therapeutic scenarios and session compositions. The results show how multimodal arts-based guidance was integrated as multisensory engagement strategies (e.g., visual art, music, and drama elements), in combination with gamification techniques. These were coupled with the development of a data-driven evaluation system. The digital therapeutic solution was refined through iterative stages of design, development, and advancement, supported by prototyping, user testing, and feedback from clinical experts, thereby addressing RQ2.

### 4.1 Key elements in the digital transformation of ACT

#### 4.1.1 Clinical evidence of ACT for depression

##### 4.1.1.1 Evidence-based therapeutic approach of ACT

ACT has been shown to effectively increase psychological flexibility and promote positive behavioral changes (39, 85). Grounded in mindfulness and Relational Frame Theory (RFT), ACT comprises six core processes—acceptance, cognitive defusion, present-moment awareness, self-as-context, values clarification, and committed action—collectively referred to as the Hexaflex model (86, 87). These processes aim to reduce psychopathology while developing adaptive psychological skills, enhancing present-moment awareness, supporting behavioral adaptation, and improving mental flexibility.

The therapeutic process integrates a range of techniques, including mindfulness practices, metaphors, experiential exercises, cognitive strategies, and behavioral activation. Clinical practice guidelines emphasize evidence-based approaches with established efficacy. Several national and international guidelines recommend ACT for depression, supported by meta-analyses and randomized controlled trials. Among them, the U.S. Veterans Affairs/Department of Defense (VA/DoD) guidelines endorse ACT—alongside Behavioral Therapy, Behavioral Activation, and CBT—as a treatment for mild to moderate major depressive disorder (88). Similarly, the Korean Academy of Medical Sciences (KAMS) and the Korea Disease Control and Prevention Agency (KDCA) recommend ACT as a first-line intervention for mild to moderate depression, citing outcomes comparable to pharmacological treatments (89).

ACT has demonstrated significant reductions in depressive symptoms across diverse clinical populations (35, 90–92), with outcomes comparable to CBT (93). These results are consistently supported by validated assessment tools such as the Beck Depression Inventory (BDI), the Patient Health Questionnaire-9 (PHQ-9), and the Center for Epidemiologic Studies Depression Scale (CES-D) (94). In addition, meta-analyses further reinforce these findings. Bai et al. (35) and Zhao et al. (36) reported moderate treatment effects of ACT in individuals with major depressive disorder. Additionally, López-Pinar et al. (37) found that ACT significantly reduces both depressive and anxiety symptoms in adolescents while enhancing psychological flexibility, the central mechanism of ACT.

Advancements in digital technology have facilitated the development of internet-delivered ACT (iACT), enabling broader access beyond traditional in-person settings. iACT is delivered through bibliotherapy, mobile applications, and web-based platforms (95–97). Meta-analytic evidence indicates that iACT, particularly for mild to moderate depression, yields small but statistically significant reductions in depressive and anxiety symptoms compared to waitlist controls, with some studies reporting comparable effects to active interventions (98, 99). iACT also contributes to improvements in psychological flexibility, confirming the adaptability of ACT’s therapeutic mechanisms to digital formats (99, 100). While face-to-face ACT demonstrates long-term effects lasting up to 19 months (91, 101), the durability of iACT outcomes remains uncertain and requires further investigation. Nonetheless, iACT is increasingly recognized as a scalable, cost-effective, and accessible intervention.

Taken together, ACT—including its digital interventions—is firmly established as an evidence-based treatment for mild to moderate depression. Although iACT tends to yield smaller effect sizes than in-person delivery, its clinical value is underscored by its reach, fidelity, and potential for integration across diverse healthcare settings. These findings provide a strong foundation for developing digital transformation strategies that extend the accessibility and consistency of ACT-based interventions.

##### 4.1.1.2 Digital transformation for ACT

This study undertook a systematic analysis of existing clinical ACT protocols to enable their adaptation into VR formats as part of the digital transformation process. We first obtained the ACT manual from the clinical team, which included nine sessions structured according to the Hexaflex model. The session composition and therapeutic structure of the original protocols were then analyzed using the SSS model as a framework. **Table 4** outlines the digital transformation strategy developed through this framework-based analysis.

**Table 4:** Digital transformation strategy for ACT based on the SSS model.

Session Structure System (SSS)		Traditional Method (TM)	Digital Transformation (DT)
Comprehensive whole sessions	Clinical period	9 sessions	5 sessions
	Goal	Enhance psychological flexibility using the Hexaflex model of ACT	
	Clinical activity	- Mindfulness - Metaphors and experiential techniques - Cognitive techniques - Behavioral activation techniques	- Gamified interactive VR experience based on ACT metaphors - Multisensory VR activities integrating multimodal arts guidance - Therapeutic narratives embedding ACT metaphors
	Evaluation method	Analysis of psychological assessment scales and questionnaires (pre-post)	Analysis of clinical and interaction-based data as objective indicators of depression
Respective single sessions	Progress structure	3 to 4 steps (introduction, core intervention, closing)	Same as TM
	Runtime	50 to 60 minutes	6 to 12 minutes depending on user engagement
	Clinical environment	Safe psychological therapy room (hospital setting)	Immersive and safe VR environment simulating real-life contexts

###### 4.1.1.2.1 Comprehensive whole sessions

To enhance therapeutic efficacy in the VR format, the original nine ACT sessions were restructured into five, integrating multisensory engagement, gamification, and therapeutic metaphors. The core therapeutic aim—to enhance psychological flexibility based on the Hexaflex model—was retained. Each session targeted specific psychological processes: acceptance, cognitive defusion, present-moment awareness, self-as-context, values clarification, and committed action.

Traditional clinical activities were reimagined as interactive, gamified experiences, delivered in a safe and immersive VR environment that stimulated multiple senses. The therapeutic scripts were carefully designed to embed ACT metaphors throughout the session flow, ensuring alignment with the underlying therapeutic framework. A core concept of this transformation—multimodal arts guidance—refers to the integration of sensory-driven artistic elements such as drawing, music, and role-playing into both the narrative and interactive structure. These elements were employed as central tools in shaping user experience and facilitating therapeutic immersion. In addition, we identified objective indicators of depression and developed a systematic evaluation framework to support their use in DTx.

###### 4.1.1.2.2 Respective single sessions

At the individual session level, each VR module followed a standardized three to four-step progression: opening, main activity, closing. The original 50–60-minute session duration was adapted to a condensed format of 6–12 minutes to suit the interactive and immersive nature of VR. These restructured sessions were designed to integrate multimodal artistic stimuli and meaningful user interactions to elicit measurable therapeutic effects. **Table 5** reorganizes the five interactive VR sessions according to the structure and themes of the original ACT manual.

**Table 5:** Reorganized ACT sessions for DTx-ACT.

Traditional manual	VR session composition			
Existing ACT manual	Track	Session	ACT keyword	Clinical goal
1. Mind welcome 2. Be here and now	Understanding myself	1	Acceptance	Psychological flexibility
3. Thoughts are just thoughts!		2	Defusion	
4. Arms wide open willingly 5. Staying with happiness		3	Present	
6. Observing self	Being together with the world	4	Self as context	
7. Life worth living 8. Are you willing to go for it? 9. Journey of life		5	Values, Committed action	

#### 4.1.2 Interactive VR integrating gamification and multimodal arts

Recent advancements in display technology have enabled digital devices to support 4K resolution or higher, facilitating the development of high-fidelity 3D environments. In VR contexts, visual stimuli are conveyed through virtual backgrounds, objects, and effects, while immersive audio components engage the auditory system via music and sound design. Musical elements within VR soundscapes are particularly essential for enhancing sensory immersion. In parallel, tactile feedback—enabled by haptic mechanisms such as controller vibrations—offers users a physical sense of interaction. Commercial VR systems currently provide basic haptic responses by adjusting vibration intensity. While these technologies form the foundational infrastructure for sensory interactions in VR, delivering immersive therapeutic experiences requires deeper content-level integration of artistic and multimodal dimensions.

Building upon this technological foundation, the present study incorporated multimodal arts—including visual, auditory, and tactile inputs—into an interactive VR therapeutic system (DTx-ACT). By embedding artistic modalities and evidence-based techniques into the system’s UI/UX design, we constructed a therapeutic environment that extends beyond conventional VR applications. This multimodal approach was operationalized through two core layers: an aesthetically driven, sensory interface and interactive user engagement based on arts-integrated tasks.

Gamification principles were systematically embedded within the interactive VR components to enhance motivation and user engagement. Specifically, key gamified features were derived from four analytical domains—user characteristics, environmental conditions, system design, and process adaptation—and structured accordingly. Whereas multimodal arts enriched the affective and sensory atmosphere through artistic media, the gamification framework introduced motivational structures such as goal orientation, feedback loops, and reward mechanisms. This dual-framework design facilitated the development of a VR-based therapeutic platform that is both emotionally resonant and behaviorally activating. The integration of these complementary strategies—multimodal arts for affective engagement and gamification for motivational reinforcement—formed the conceptual foundation of the DTx-ACT system. A detailed summary of the gamification features incorporated into the system is presented in **Table 6**.

**Table 6:** Analysis of gamification elements integrated into interactive VR components within the DTx-ACT system.

Gamification principles	Interactive VR elements	Features
User characteristics	Depression	• Diagnosis and symptoms: depression • Clinical goal: psychological flexibility
Environment	VR	• 360-degree 3D space: multisensory and synesthetic stimulation • User interface (UI): aesthetics (look and feel) • User experience (UX): immersive and interactive activity • Data collection: interaction data (sensor and log)
System design	Multimodal arts guidance	• Service scenario: digital storytelling • Storyboard and script for visualization (UI): - Graphic (background images, objects) - Sound (background music, sound effects) - Direction (dialogue, action, setting, blocking, props, captions) • Interactive arts activities (UX): - Techniques from art, music, drama - Multisensory stimulation (sight, sound, tactile feedback)
Translational	Digital transformation of ACT	• Therapeutic session structure: the SSS model: - Comprehensive whole sessions - Respective single sessions • Therapeutic process: the Hexaflex model - Acceptance - Defusion - Present moment awareness - Self as Context - Values - Committed Action • Therapeutic interventions via metaphors: - Voice-guided therapeutic instructions - Arts-guided therapeutic interactions

##### 4.1.2.1 User characteristics

DTx-ACT was designed to alleviate depressive symptoms by enhancing psychological flexibility. Throughout the development of the therapeutic system, the characteristics of individuals with depression were carefully considered.

##### 4.1.2.2 Environment

The therapeutic environment was developed using VR elements to support immersive engagement. The VR setting offered realistic, multisensory content that enhanced synesthetic experiences. Spatial exploration involved not only understanding one’s surroundings but also managing potential threats within the environment. Through immersive technologies, a 360-degree, three-dimensional space was created to deeply engage users and promote a heightened sense of presence and realism. Accordingly, the VR system was designed to facilitate active user interaction with the virtual space. Furthermore, the UI/UX were carefully developed to enable users to interact with and control the environment with greater autonomy.

##### 4.1.2.3 System design

The system was designed to integrate multimodal arts guidance into the UI/UX. Guided by digital storytelling principles, the development process included service scenarios, storyboards, and therapeutic scripts to visualize the treatment flow. The DTx-ACT system incorporated multisensory elements into interactive therapeutic activities.

Visual art elements were integrated into the VR design through background graphics, virtual objects, animation effects, motion imagery, color schemes, and visual guidance (e.g., mindfulness prompts), which collectively created immersive visual experiences. Haptic controllers were employed to enable drawing and coloring tasks, simultaneously stimulating both visual and tactile senses. Core enabling technologies included hand tracking, eye tracking, and 3D painting.

Music elements contributed to immersion through background music (BGM) tailored to each session and sound effects synchronized with interactive scenarios. These auditory features enhanced the immersive quality and enriched the therapeutic experience. To convey ACT-specific metaphors more effectively, music interaction was developed, incorporating haptic feedback triggered by object-touch events.

Dramatic and literary elements were incorporated into the therapeutic scripts and overall scene composition. Drama emphasized roleplay and narrative structure to create immersive and projective experiences that bridged virtual and real-world contexts. These components served as the foundation for therapeutic storytelling and ACT-related metaphors. For example, users interacted with avatars and followed narrative perspectives, while metaphor-based tasks—such as avatar customization and the hero’s journey—were applied to restructure therapeutic content. Literary components were implemented through linguistic guidance, including subtitles and voice prompts. Additionally, speech-to-text (STT) functionality allowed users to articulate their thoughts, supporting expressive techniques and active participation.

Dance and movement elements emphasized spatial recognition and embodied awareness within the VR environment rather than overt physical activity. Interaction tasks—such as touching and clicking using haptic controllers—naturally elicited body movement during creative activities like drawing and music interaction. These experiences promoted somatic engagement by integrating spatial perception, bodily gestures (e.g., motion sensors), and dynamic visual elements, thereby enhancing physical activation and the sense of presence.

##### 4.1.2.4 Translational

Finally, gamification was applied as a key translational strategy in the digital transformation of ACT, bridging clinical principles with interactive user experiences. In conjunction with multimodal arts elements, gamification served to restructure therapeutic sessions based on the SSS model and to guide the therapeutic journey through the Hexaflex framework. The intervention design was grounded in ACT metaphors, which were delivered through voice-guided instructions and arts-based therapeutic interactions.

#### 4.1.3 Data-driven evaluation system

##### 4.1.3.1 Conceptual foundation of behavioral and sensor indicators for depression

Depression assessment has progressed through the incorporation of behavioral signals and sensor technologies, providing objective data to augment traditional diagnostic methods. Recent research demonstrates that functional features of depression, including specific behavioral characteristics and sensor-derived biomarkers, can be effectively employed for automated detection of depressive symptoms (102–104).

Behavioral patterns observed in individuals with depression reveal significant differences compared to those of healthy controls, notably in head movements, eye movements, facial expressions, and vocal acoustic features. For instance, individuals with depression tend to exhibit slower head movements, fewer changes in head position, and longer durations of gaze directed downward or to the right, which may suggest fatigue or avoidance of eye contact (105). These head and eye movement patterns have been identified as effective biomarkers for detecting depressive symptoms (106). Facial expressions also serve as significant biomarkers of depression, with affected individuals displaying more frequent negative expressions and fewer positive ones. Research further highlights attention bias as a key marker, demonstrating a tendency to focus more on negative emotional stimuli than on positive ones (107). Moreover, speech features—such as slower speech rates, reduced pitch variation, and longer pause durations—further distinguish depression (108). **Table 7** provides an overview of these functional biomarkers identified in existing studies.

**Table 7:** Functional features of depression reflected in behavioral and sensor-based biomarkers.

Biomarkers category	Functional features of depression	References
Head Movement	• Slower head movements • Reduced head position variability	(105)
Eye Movement	• Longer gaze duration to the right • Prolonged downward gaze, indicating fatigue and avoidance of eye contact	(105)
	• Longer fixation durations • Slower saccadic eye movements • Increased eye contact avoidance, especially in response to negative emotional expressions	(109)
	• Larger leftward attentional bias, indicating right hemisphere overactivation during facial expression recognition • More fixations on the left visual field of the face	(110)
	• Fewer and shorter fixations on positive emotional stimuli • More and longer fixations on negative emotional stimuli • Decreased attentional bias toward positive information, accompanied by increased bias toward negative information	(107)
Facial Expression	• Decreased activity in the muscles involved in smiling, particularly the zygomaticus major	(111)
	• Lower accuracy and longer reaction times in facial expression recognition tasks	(112)
Vocal Acoustic	• Slower speech rate • Reduced pitch variation • Longer pause durations	(108)

Building on this body of evidence, we identified core data types suitable for real-time acquisition within a VR environment. The Meta Quest Pro device was selected to capture sensor data, including head movements, eye-tracking, facial expressions, and vocal acoustic features—key indicators aligned with validated depression-related biomarkers.

##### 4.1.3.2 Feasibility of multimodal indicators for the DTx-ACT system: a pilot study

Building on the conceptual framework of behavioral and sensor indicators, we tested their feasibility in a pilot study using a three-session VR-MBCT prototype (82). Participants were assigned to either the individuals with depression (IWD; n = 38) or individuals without depression (IWoD; n = 35) group based on PHQ-9 scores.

Both groups demonstrated high attentional engagement, with an average gaze fixation rate of approximately 88%, and no session recording less than 83%. No significant group differences were observed, indicating comparable levels of visual attention and immersion. In contrast, distinctive behavioral patterns were identified across sessions.

In the constellation-drawing task (Session 1), the IWD group revisited individual stars significantly more frequently (M = 3.62, SD = 3.53) than the IWoD group (M = 0.81, SD = 1.67), F(1,65) = 14.77, p = 0.0003, η^2^_p_ = 0.19. The total number of revisits was also significantly higher in the IWD group (M = 7.78, SD = 7.39) than in the IWoD group (M = 0.89, SD = 2.11), F(1,65) = 29.66, *p* < 0.001, η^2^_p_ = 0.31. These findings suggest more repetitive and exploratory interaction patterns among individuals with depressive symptoms.

In the avatar selection task (Session 2), the IWD group exhibited significantly longer response latency when choosing an avatar to represent their current state (M = 80.83 sec, SD = 27.08) compared to the IWoD group (M = 55.29 sec, SD = 10.49), F(1,65) = 24.53, p < 0.001, η^2^_p_= 0.27. The IWD group also showed a broader and more skewed distribution of response times, indicating increased hesitation or cognitive effort.

In the emotion-selection task (Session 3), significant group differences were observed in both positive and negative emotion choices. The IWD group selected fewer positive emotions (M = 2.22, SD = 2.58) than the IWoD group (M = 4.17, SD = 3.66), F(1,65) = 4.01, p = 0.05, η^2^_p_ = 0.06. Conversely, the IWD group selected more negative emotions (M = 4.38, SD = 3.29) than the IWoD group (M = 1.33, SD = 1.17), F(1,65) = 29.39, p < 0.001, η^2^_p_ = 0.31. These results align with established emotional biases in depression and support the use of affective interaction data as evaluative indicators.

Additionally, the IWD group exhibited significantly higher electrodermal activity (EDA) entropy (M = 5.77) than the IWoD group (M = 5.02), p = 0.04. Although this physiological indicator demonstrated discriminative potential, it was excluded from the final DTx-ACT system due to limitations in real-time processing.

In summary, this pilot study validated gaze fixation, behavioral responses, and sensor-based data as feasible and reliable indicators for assessing user engagement and emotional response in immersive VR environments. These findings directly informed the design of the data-driven evaluation framework embedded in the DTx-ACT system.

### 4.2 Development of interactive VR-based DTx for ACT (DTx-ACT)

#### 4.2.1 Service scenario design

##### 4.2.1.1 Session 1: Myself inside the mirror

The first session, “Myself inside the mirror,” consisted of three key components: (a) mindfulness, (b) assisting an avatar in distress, and (c) acceptance through emotional labeling. Guided meditation and interactive tasks were implemented to relax the autonomic nervous system and foster non-judgmental awareness. The session aimed to strengthen ego functioning by promoting self-acceptance, thereby enhancing individuals’ capacity to adapt to environmental changes and cope with stress. The design of this session was grounded in established clinical evidence.

##### 4.2.1.2 Session 2: Whispers of the mind

The session, “Whispers of the mind,” consisted of three stages: (a) self-reflection, (b) externalizing the voice of the mind, and (c) listening to music. Individuals with depression tend to over-identify with negative thoughts and emotions. To address this, defusion techniques were incorporated to help participants detach negative evaluations from their self-identity and reconstruct a more adaptive self-concept. Auditory stimuli were used to enhance sensory engagement, given that individuals with depression often show diminished responsiveness to external inputs. This design was informed by clinical evidence underscoring the therapeutic benefits of sensory stimulation.

##### 4.2.1.3 Session 3: Hues of emotion

The session, “Hues of emotion,” consisted of four stages: (a) mindfulness breathing, (b) present-moment awareness, (c) spatial orientation within the environment, and (d) reflective sharing. Individuals with depression often exhibit an attentional bias toward negative emotional stimuli, which contributes to persistent rumination. To counter this, the session incorporated multimodal art-based guidance using visual stimuli to redirect attention to the present moment and cultivate an immersive “here-and-now” experience. By disrupting ruminative cognitive patterns and enhancing present-centered awareness, the session aimed to help participants process external stimuli more objectively and with greater emotional balance. This design was informed by clinical findings emphasizing the therapeutic value of sensory engagement.

##### 4.2.1.4 Session 4: Yourself inside the mirror

The session, “Yourself inside the mirror,” consisted of three activities: (a) creating a personal avatar, (b) listening to the avatar’s concerns, and (c) practicing empathy and providing comfort. Individuals with depression often exhibit negative biases toward themselves, others, and the world. To address this, the session applied the “empty chair” technique to help participants project their concerns and emotions onto the avatar, fostering self-objectification and supporting cognitive reevaluation. This design was informed by clinical evidence highlighting the therapeutic value of externalizing and reframing personal experiences.

##### 4.2.1.5 Session 5: A novel self

The session, “A novel self,” consisted of three phases: (a) identifying personal values and life goals, (b) embarking on a metaphorical journey toward these goals, and (c) reaching the destination and reviewing a checklist of achievements. Individuals with depression often experience diminished motivation, which hinders effective goal-setting and follow-through. To address this, the session guided participants in clarifying core values and engaging in committed actions through a metaphorical, goal-oriented journey. Additionally, attention-shifting tasks were incorporated to enhance cognitive flexibility, targeting the attentional narrowing and difficulties with transitioning focus that are commonly observed in depression. This design was informed by clinical evidence emphasizing the therapeutic impact of value-based action and attentional control.

#### 4.2.2 Visualization of therapeutic sequences

##### 4.2.2.1 Considerations for VR-based therapeutics

The content was carefully designed to ensure psychological safety and emotional sensitivity. These features supported the therapeutic efficacy of VR-based interventions and enhanced user experience. The development of VR-based therapeutics required deliberate consideration of factors that ensured user safety while promoting immersive engagement. For instance, high-speed visual stimuli were adjusted to avoid discomfort or aversion in users with depressive symptoms. Similarly, enclosed or overly dark virtual spaces were excluded to reduce anxiety responses. Instead, dreamlike and surreal landscapes were employed to foster psychological calmness and emotional relief. Sudden visual or auditory effects, as well as elements that might trigger excessive fear, were systematically removed to prevent negative experiences. Clinical experts were closely involved throughout the development process to guide these considerations. As a result, safe, immersive, and interactive components were successfully embedded into the therapeutic activities and virtual environments of DTx-ACT.

##### 4.2.2.2 Implementation of therapeutic sequences of DTx-ACT

The interactive VR system developed in this study was implemented in accordance with a therapeutic design framework, with a particular focus on gamification principles to promote full engagement in artistic activities. Each session was realized as immersive, interactive VR content that embodied the core therapeutic mechanisms of ACT. The system was intended to enhance emotional regulation and alleviate depressive symptoms through targeted therapeutic experiences.

**Figure 1:**
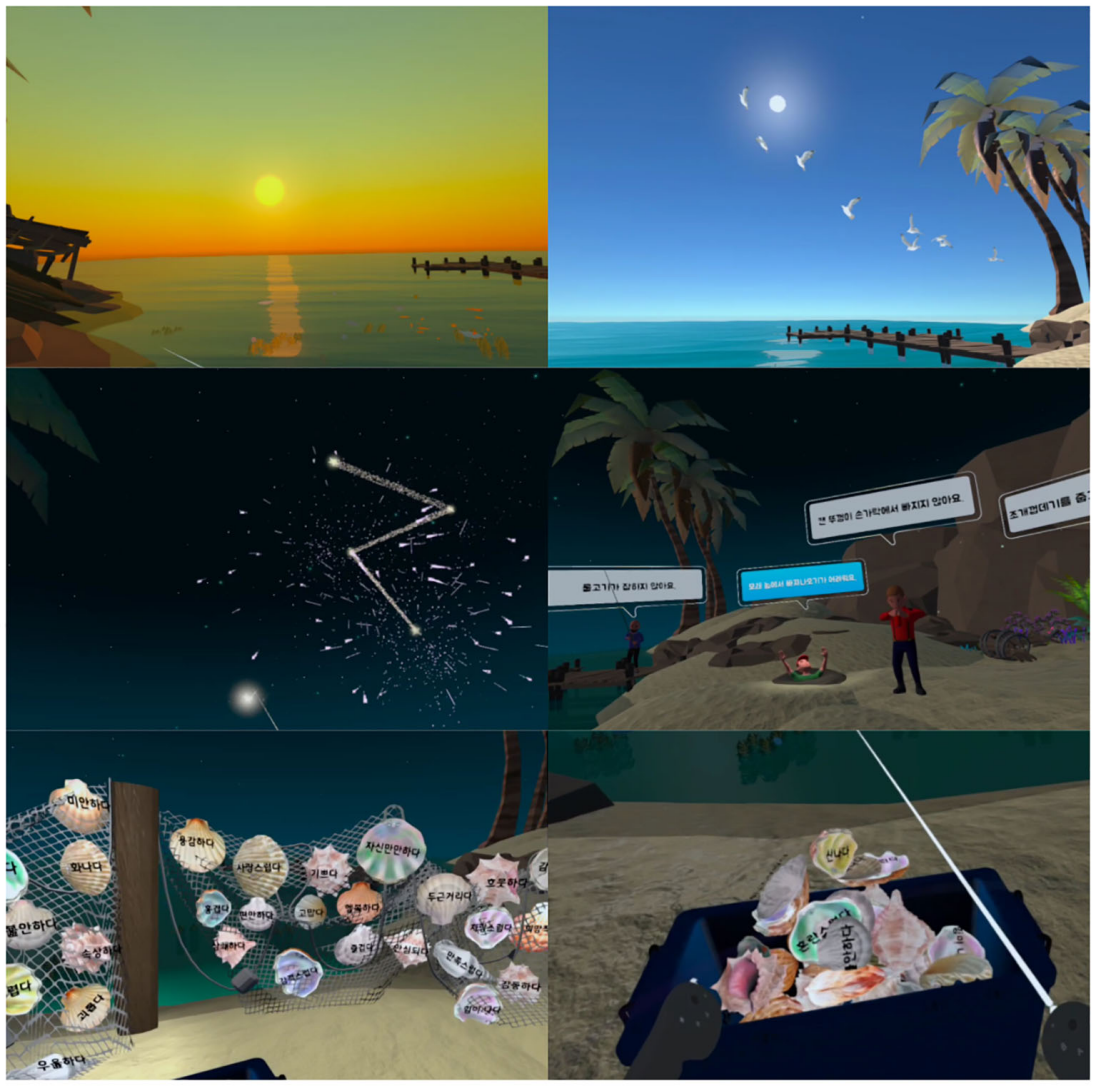
Visualization of session 1: Myself inside the mirror.

Session 1, “Myself inside the mirror,” featured mindfulness interactions that focused on the acceptance of experiences (**Figure 1**). Meditation and metaphorical content enabled participants to accept various emotions and thoughts, fostering a non-judgmental awareness of their inner experiences.

**Figure 2:**
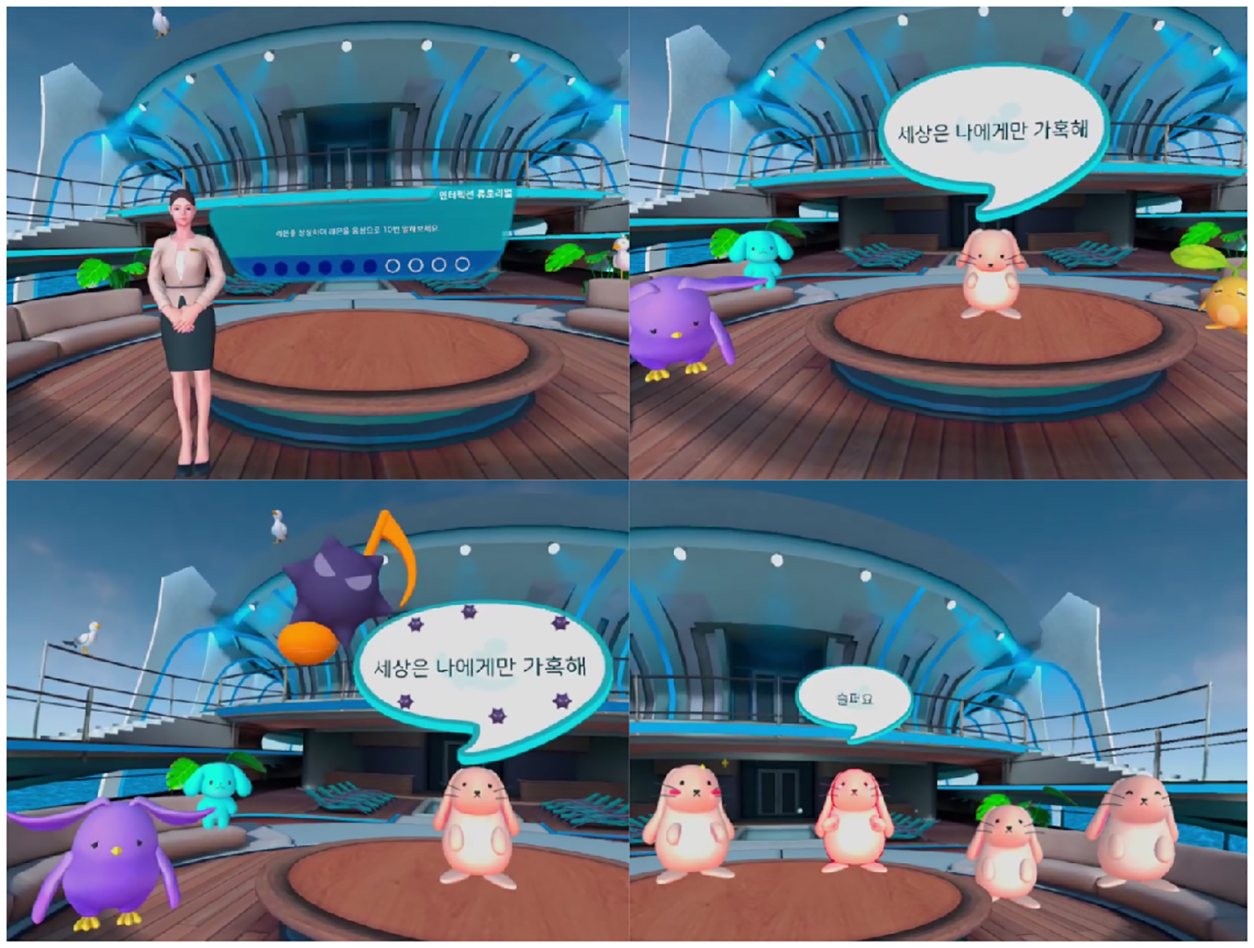
Visualization of session 2: Whispers of the mind.

Session 2, “Whispers of the mind,” included music interactions focusing on defusion (**Figure 2**). Through metaphorical content composed of visual and auditory guidance, participants experienced a symbolic disconnection between their negative or biased self-image and their real or objective self-concept.

**Figure 3:**
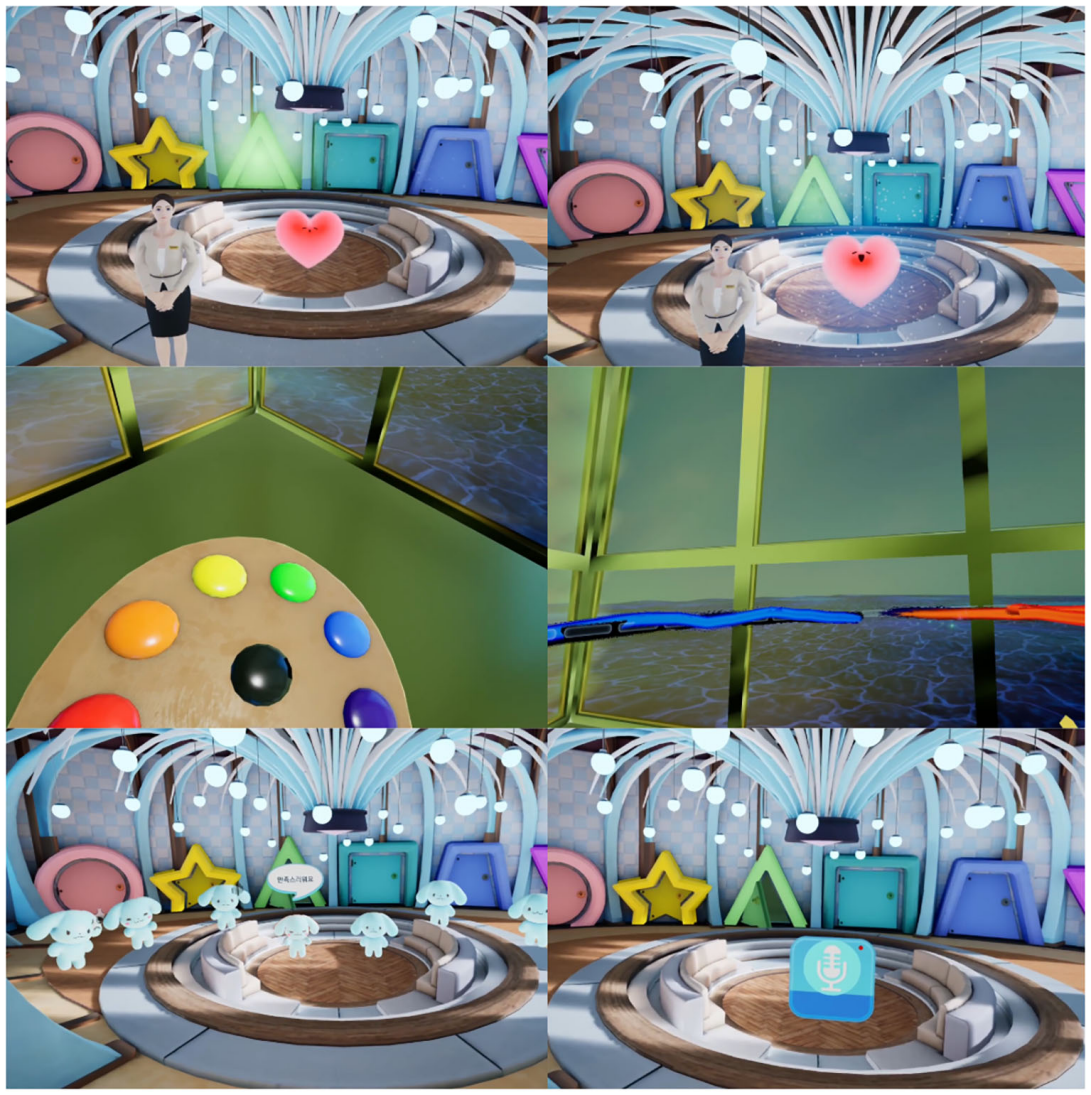
Visualization of session 3: Hues of emotion.

Session 3, “Hues of emotion,” included drawing interaction focused on connecting with the present moment (**Figure 3**). This content addressed the tendency of patients with depression to ruminate on the past and fixate on negative events. Through dynamic, multisensory drawing activities, participants engaged in immersive “here and now” experiences.

**Figure 4:**
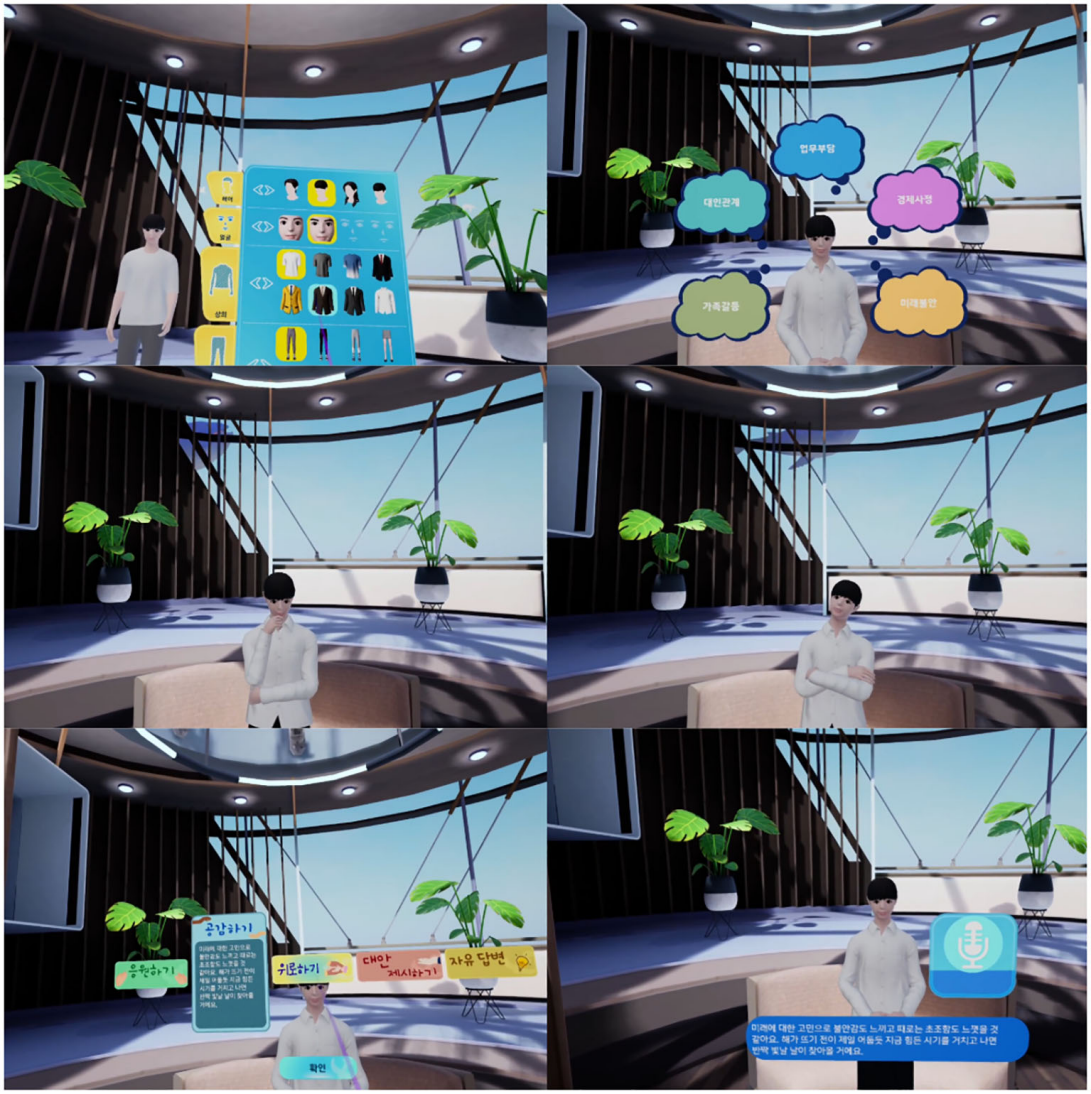
Visualization of session 4: Yourself inside the mirror.

Session 4, “Yourself inside the mirror,” included dramatic interactions that focused on the self as context (**Figure 4**). Based on the empty-chair technique, this therapeutic approach enabled participants to digitally reimagine role-playing and role training, supporting self-objectification and cognitive reappraisal.

**Figure 5:**
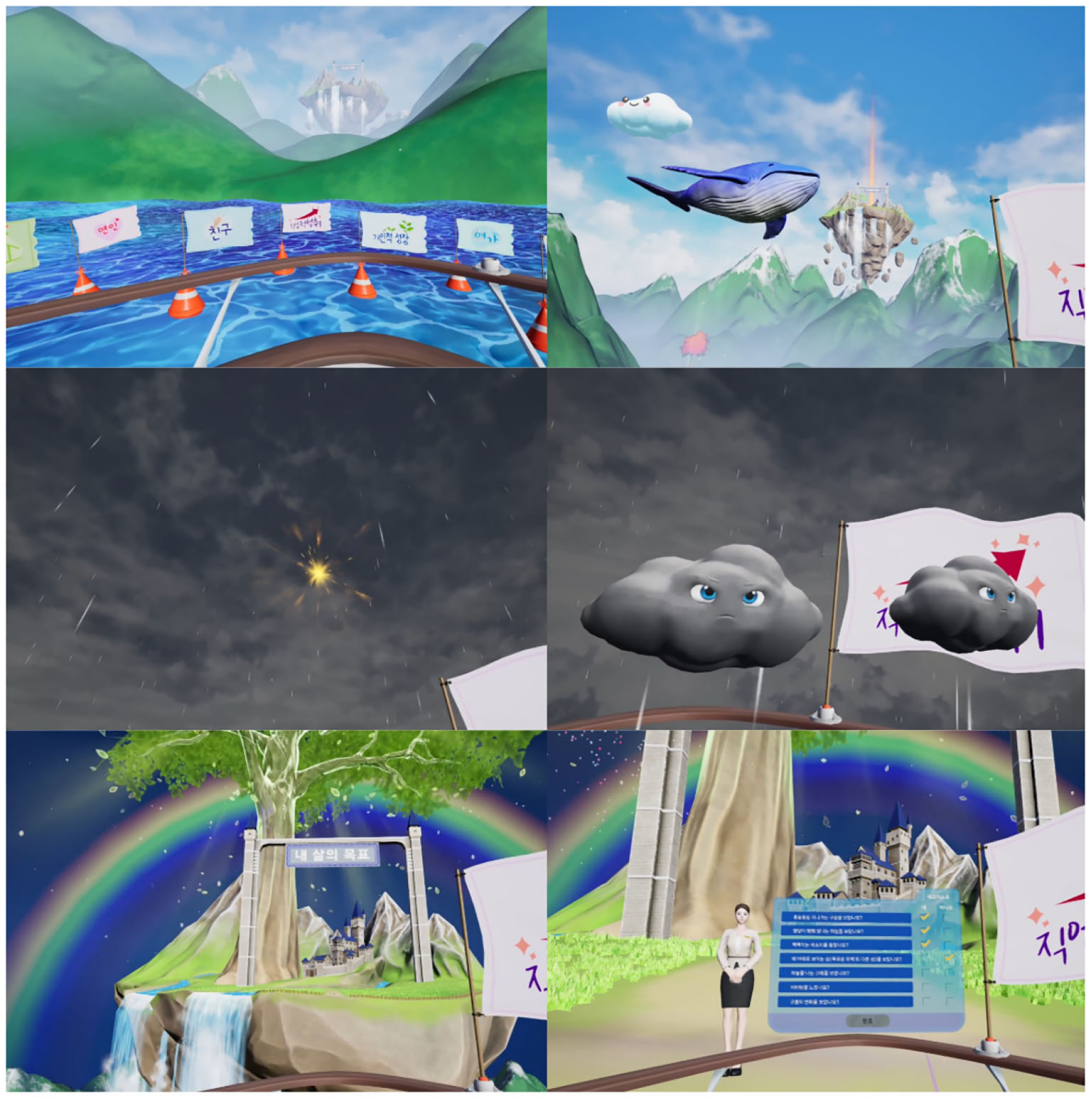
Visualization of session 5: A novel self.

Session 5, “A novel self,” included dramatic interactions that focused on values and committed actions (**Figure 5**). This session guided participants through a hero’s journey narrative, enabling them to clarify personal values and pursue life goals, thereby helping them overcome apathy, lethargy, and inactivity.

#### 4.2.3 Implementation of the data-driven evaluation system

##### 4.2.3.1 System architecture and data flow of DTx-ACT

The system architecture of DTx-ACT provides a structural framework for the automated collection and processing of multimodal user data during VR-based therapeutic sessions. It supports key functions, including sequential content delivery, in-session interaction logging, and engagement monitoring. The architecture also outlines pathways for integrating real-time data and enabling interoperability with clinical systems in future applications.

To facilitate immersive engagement in multimodal arts-based activities, the system incorporates ACT-informed guidance scenarios and interactive scripts. During each session, it collects both sensor-based data (e.g., gaze tracking, hand positioning) and interaction logs to assess user attention, immersion, and task performance. These data streams can be automatically processed to monitor protocol adherence and evaluate user engagement.

**Figure 6:**
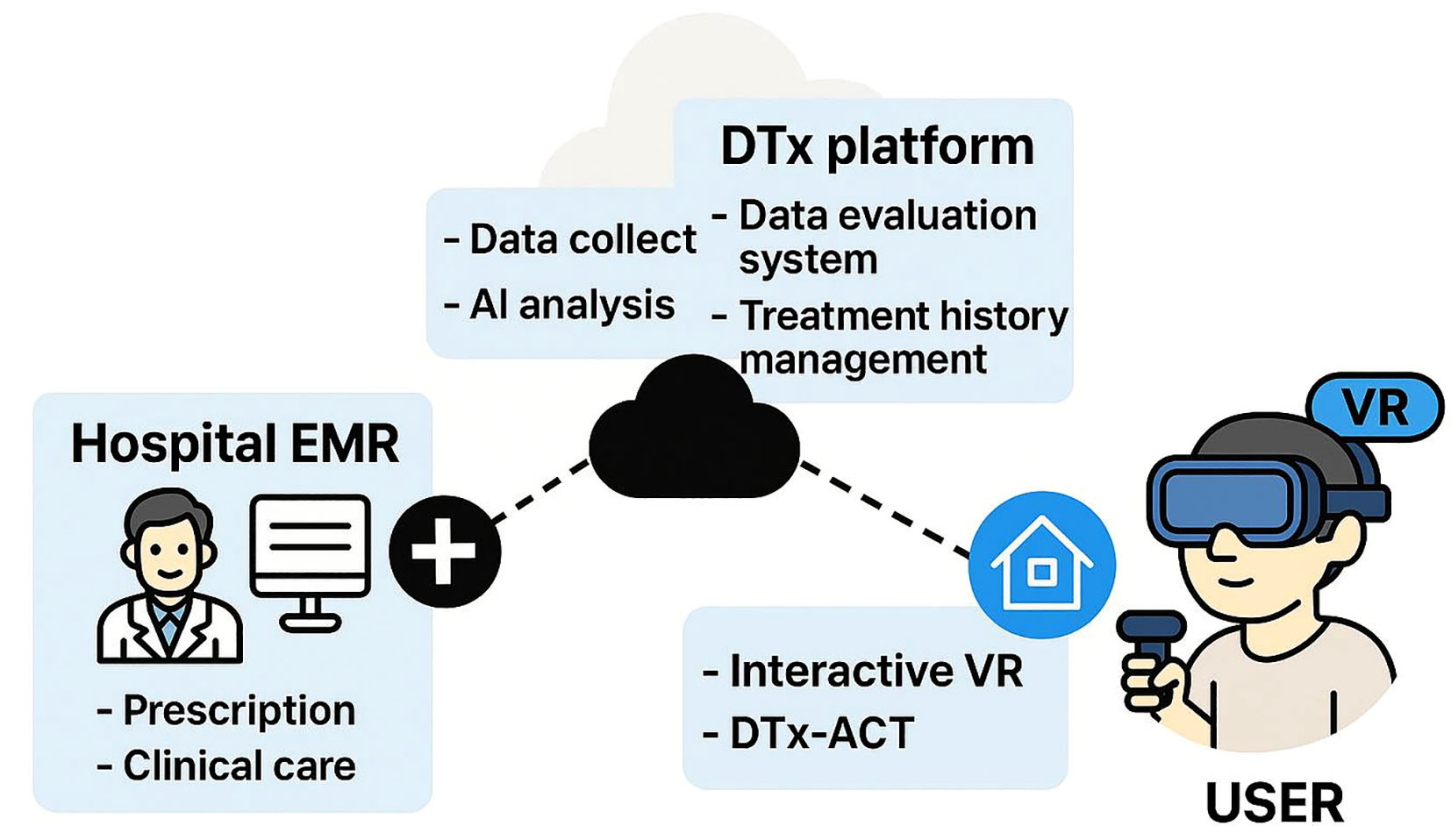
The system architecture of interactive VR-based DTx. This diagram was visualized using an AI-based generative model (OpenAI’s DALL·E) to conceptually represent the integrated system architecture of the DTx-ACT environment.

The system currently supports partially automated data capture and processing. Implemented functions include content synchronization, interaction tracking via the HMD, and internal analysis of engagement data. **Figure 6** illustrates the system architecture and data flow of DTx-ACT.

##### 4.2.3.2 Multimodal data features and indicators for the DTx-ACT framework

Building on the system architecture described above, the DTx-ACT framework was designed to collect and integrate multimodal data streams during VR-based therapeutic sessions to evaluate depression-related symptoms and user engagement. The system is structured around two primary data types: clinical data and interaction data.

Clinical data quantify subjective changes in depression symptoms through standardized screening tools such as the PHQ-9 and pre/post VR psychological assessments. Diagnostic information and treatment history obtained from electronic medical records (EMRs) may also be included. These data serve as core indicators in the DTx-ACT evaluation system and will support future clinical validation by linking real-world clinical outcomes to interaction patterns.

Interaction data include both sensor-derived and behavioral log data, collected in real-time through HMD sensors during therapeutic sessions. Sensor metrics—such as gaze behavior, head movement, and facial expressions—are used to assess attention, psychomotor activity, and emotional responses. Log data capture task-related behaviors, including interaction latency, task completion time, and action frequency, which are used to evaluate immersion, task efficiency, and cognitive responsiveness.

A pilot study was conducted to preliminarily validate the feasibility of these multimodal indicators (82), and the results were integrated into the system’s design. In future development, the DTx-ACT framework will incorporate an AI-powered analysis pipeline optimized for VR-DTx environments. Specifically, AI models will be introduced for real-time engagement classification, behavioral pattern recognition, and anomaly detection. Through this AI-driven feedback mechanism, DTx-ACT is expected to evolve into a personalized therapeutic platform that dynamically adapts content based on user responses and immersion levels. **Table 8** summarizes the multimodal data metrics used in the DTx-ACT framework.

**Table 8:** Data matrix of clinical and interaction data types in the DTx-ACT system.

Data types	Data features	Data items
Clinical data		
**●** Screening data	Quantitative measures of depressive symptoms collected before and after VR content interaction; used to identify participants and evaluate treatment effectiveness.	- Depression and anxiety screening tests (e.g., PHQ-9, BDI-II, HAM-D).
**●** EMR data	Clinical information obtained from electronic medical records (EMRs), including patient history and medical examination results; used for comprehensive health status analysis.	- Medical history - Medication records
Interaction data		
**●** Sensor data	Movement and physiological signals collected through head-mounted displays (HMDs).	- Gaze tracking (e.g., objects within the content) - Head movement tracking (e.g., position and rotation) - Eye movement tracking (e.g., origin and direction) - Facial movement tracking (e.g., brow, cheek, chin, dimple, jaw, lid, lip, mouth, and nose wrinkle)
**●** Log data	Behavioral records generated during user interactions within VR content.	- Interaction duration data (e.g., latency to start actions, time spent on interaction) - Types of selected actions

## 5 Discussion and conclusion

### 5.1 Principal findings

This study presents a structured and replicable development framework for transforming ACT into an interactive, VR-based digital therapeutic intervention targeting depression. The proposed framework provides a theoretical and procedural foundation for the digital transformation of evidence-based psychotherapeutic models into immersive, personalized, and data-driven interventions.

The conceptual structure for digital transformation follows a comprehensive roadmap grounded in the PLM, encompassing five iterative phases: preliminary research, design, development, advancement, and commercialization. The application of the SSS model enables modular restructuring of traditional evidence-based treatment protocols, addressing both macro-level intervention structures (e.g., clinical goals, therapeutic activities, evaluation methods) and micro-level session elements (e.g., sequence, duration, environmental context).

This framework is defined by three core components:

1. The systematic structuring of clinical content grounded in the theoretical mechanisms of EBPs, such as ACT.
2. The integration of interactive features combining gamification strategies and multimodal arts—including visual art, music, drama, literature, and movement—to enhance emotional engagement and multisensory immersion.
3. The development of a real-time evaluation system that leverages behavioral logs and sensor-derived data to objectively assess therapeutic outcomes.

These components are implementable within interdisciplinary research and development environments involving digital healthcare, psychiatry, clinical psychology, human–computer interaction (HCI), data science, and arts therapy. Therapeutic scenarios and narrative structures can be designed according to gamification principles that reflect user characteristics and contextual conditions. In addition, visualization and UX elements should be continuously refined through iterative feedback and adjusted dynamically to align with therapeutic objectives and user engagement levels.

The DTx-ACT framework transcends conventional content delivery models of DTx by integrating emotionally responsive interaction design, data-driven feedback mechanisms, and real-time adaptive systems. This structure enables the development of precision-oriented, patient-centered digital mental health interventions. Accordingly, the proposed framework provides a transferable and scalable model for designing interactive digital therapeutic systems that systematically integrate clinical fidelity, immersive engagement, and quantifiable assessment across diverse mental health domains.

### 5.2 Limitation and future scope

While the proposed framework demonstrates considerable promise as an immersive digital intervention for mental health, several important limitations remain. Most notably, the system has not yet undergone empirical validation through large-scale clinical trials or real-world data analysis. Although regulatory processes were initiated in parallel with the MFDS, this system remains at the pre-commercialization stage.

In addition, the absence of integrated datasets combining EMR with VR-based interaction data constrains both the generalizability and clinical utility of the framework. Although pilot studies have identified the potential of interaction- and biosignal-based digital biomarkers, these findings remain preliminary and require further validation against established clinical indicators and outcomes. Addressing these limitations will require future studies to prioritize large-scale clinical trials involving diverse patient populations, as well as the integration of EMR data with multimodal interaction data—such as behavioral logs and sensor-derived signals from VR sessions—to generate comprehensive, data-driven insights into patient states and therapeutic responses.

To further improve system capabilities, an intelligent analytics infrastructure must be developed. This infrastructure should support real-time engagement monitoring, anomaly detection, and adaptive content delivery. Context-aware, AI-powered personalization within the VR environment may further enhance adherence and improve therapeutic outcomes. Furthermore, achieving seamless interoperability among the DTx-ACT platform, EMR systems, and other digital therapeutic infrastructures is essential. Such integration will support the incorporation of multimodal data into clinical workflows, enhance evidence-informed clinical decision-making, and contribute to the advancement of precision mental health care.

## 6 Conclusions

This study presents a structured framework for developing an interactive VR-based DTx system, grounded in a digital transformation approach that integrates gamification and multimodal arts. The framework is designed to translate ACT into immersive, technology-enabled mental health interventions. By supporting emotionally engaging and patient-centered therapeutic experiences while maintaining clinical fidelity, it enhances user participation and sustained immersion through interactive, modular session designs tailored to digital environments.

A key contribution of this study is the development of a data-driven evaluation system that leverages sensor-based responses and arts-based interactions as potential digital biomarkers. This approach provides theoretical and practical insights into the personalization and precision of mental health interventions. The framework is reproducible and scalable, enabling its transferability across diverse clinical settings and adaptability to a wide range of mental health conditions beyond depression. By balancing clinical theory with user engagement science, it addresses notable limitations in existing DTx through a creatively enriched and practically grounded development model.

Furthermore, the study conceptualizes arts-based interaction as a quantifiable therapeutic signal, expanding the scope of digital biomarkers beyond physiological sensing to include affective responses guided by multimodal arts. This reinforces the clinical validity of arts-based therapeutic mechanisms and opens new directions for emotionally precise, personalized care. Ultimately, the proposed framework lays a foundation for next-generation mental health interventions that are immersive, interactive, evidence-based, and clinically actionable—marking a significant contribution to the advancement of precision-driven DTx.

## Data Availability

The original contributions presented in the study are included in the article/supplementary material. Further inquiries can be directed to the corresponding author.
